# Clinical consequences and cost of limiting use of vancomycin for perioperative prophylaxis: example of coronary artery bypass surgery.

**DOI:** 10.3201/eid0705.010508

**Published:** 2001

**Authors:** G Zanetti, S J Goldie, R Platt

**Affiliations:** Channing Laboratory, Brigham and Women's Hospital, and Eastern Massachusetts CDC Prevention Epicenter, Boston, USA. Giorgio.Zanetti@chuv.hospvd.ch

## Abstract

Routine us of vancomycin for perioperative prophylaxis is discouraged, principally to minimize microbial resistance to it. However, outcomes and costs of this recommendation have not been assessed. We used decision-analytic models to compare clinical results and cost-effectiveness of no prophylaxis, cefazolin, and vancomycin, in coronary artery bypass graft surgery. In the base case, vancomycin resulted in 7% fewer surgical site infections and 1% lower all-cause mortality and saved $117 per procedure, compared with cefazolin. Cefazolin, in turn, resulted in substantially fewer infections and deaths and lower costs than no prophylaxis. We conclude that perioperative antibiotic prophylaxis with vancomycin is usually more effective and less expensive than cefazolin. Data on vancomycin's impact on resistance are needed to quantify the trade-off between individual patients' improved clinical outcomes and lower costs and the future long-term consequences to society.


### Clinical Consequences and Cost of Limiting Use of Vancomycin for Perioperative Prophylaxis: Example of Coronary Artery Bypass Surgery

**Giorgio Zanetti,*† Sue J. Goldie,‡ and Richard Platt*§**

*Channing Laboratory, Brigham and Women's Hospital, and Eastern Massachusetts CDC Prevention Epicenter, Boston, Massachusetts, USA; †University Hospital, Lausanne, Switzerland; ‡Harvard School of Public Health, Boston, Massachusetts, USA; and §Harvard Medical School and Harvard Pilgrim Health Care, Boston, Massachusetts, USA

Routine use of vancomycin for perioperative prophylaxis is discouraged, principally to minimize microbial resistance to it. However, outcomes and costs of this recommendation have not been assessed. We used decision-analytic models to compare clinical results and cost-effectiveness of no prophylaxis, cefazolin, and vancomycin, in coronary artery bypass graft surgery. In the base case, vancomycin resulted in 7% fewer surgical site infections and 1% lower all-cause mortality and saved $117 per procedure, compared with cefazolin. Cefazolin, in turn, resulted in substantially fewer infections and deaths and lower costs than no prophylaxis. We conclude that perioperative antibiotic prophylaxis with vancomycin is usually more effective and less expensive than cefazolin. Data on vancomycin's impact on resistance are needed to quantify the trade-off between individual patients' improved clinical outcomes and lower costs and the future long-term consequences to society.

The emergence of vancomycin-resistant enterococci has opened a new era of hardly treatable bacterial infections, and there is now evidence that more virulent common pathogens such as *Staphylococcus aureus* can also develop resistance to vancomycin (1,2). The use of vancomycin is hypothesized to promote the development or transmission of this resistance (3,4). Restrictive guidelines have therefore been disseminated for the use of vancomycin or teicoplanin, another glycopeptide agent (5). These guidelines include a recommendation against the routine use of vancomycin as perioperative antibiotic prophylaxis for surgical site infections.

However, vancomycin is preferred for preventing infections caused by methicillin-resistant *Staphylococcus aureus* (MRSA) or methicillin-resistant coagulase-negative staphylococci. This is the rationale for recommending vancomycin prophylaxis when the risk for infection from methicillin-resistant pathogens is high (6-11), although no guideline has made a clear statement on when to use this alternative. Since antibiotics are commonly used for prophylaxis, liberal interpretation of the prophylaxis guidelines will clearly jeopardize efforts to limit the use of vancomycin. Vancomycin is also more expensive to purchase and administer than cephalosporins.

To inform both the clinical and public policy debate with respect to the optimal prophylaxis regimen, we conducted a cost-effectiveness analysis to compare the short- and long-term consequences of using vancomycin and cefazolin as first-line perioperative prophylaxis. We focused on patients who underwent coronary artery bypass graft surgery (CABG) because this is a large, relatively homogeneous population with substantial risk for serious surgical site infection (12,13).

Address for correspondence: Giorgio Zanetti, Division of Infectious Diseases, University Hospital, 1011 Lausanne, Switzerland; fax: 4121-314-1018; e-mail: Giorgio.Zanetti@chuv.hospvd.ch

### Methods

### Cost-Effectiveness Analysis

We developed a decision-analytic model (Figure 1) to calculate the clinical benefits and costs associated with alternative strategies for antibiotic prophylaxis in a hypothetical cohort of 10,000 patients undergoing CAGB surgery. The three strategies evaluated were 1) no prophylaxis; 2) routine cefazolin, reserving vancomycin for those with a history of allergic reaction to beta-lactam antibiotics; and 3) routine vancomycin. In the base-case analysis, we adopted a payer perspective and included clinical outcomes and direct medical costs in the 3 months after surgery. Clinical outcomes included deep and superficial surgical site infections, as well as hospital deaths.

We also conducted a reference case analysis, as recommended by the Panel on Cost-Effectiveness in Health and Medicine (14), which assumed a societal perspective and relied on a longer time horizon. The reference case was a 65-year-old man undergoing CABG surgery for stable multivessel coronary heart disease. A state-transition model incorporated the lifetime probability of death, myocardial infarction, angina, or asymptomatic coronary artery disease following CABG surgery (15,16) to estimate life expectancy, quality-adjusted life expectancy, and total lifetime costs. Future costs and benefits were discounted at an annual rate of 3%.

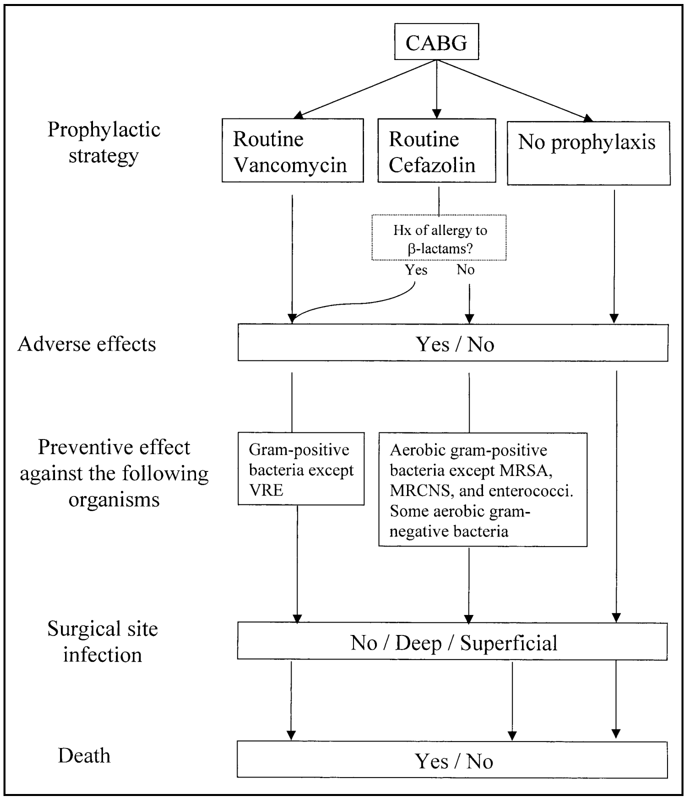

Figure 1. Model of the decision tree.*
*CABG = coronary artery bypass graft surgery; Hx = history of; MRSA = methicillin-resistant *Staphylococcus aureus;* MRCNS = methicillin-resistant coagulase-negative staphylococci; VRE = vancomycin-resistant enterococci.

To conduct the cost-effectiveness analysis, the three strategies were ranked by increasing effectiveness; those that cost more but were less effective than an alternative strategy were eliminated by strong dominance. When one strategy was more effective but more costly, an incremental cost-effectiveness ratio was calculated by dividing the additional cost of this specific strategy by its additional clinical benefit, compared with the next least expensive strategy. We conducted uni- and multivariate sensitivity analyses to assess the stability of the results in the face of plausible variation in the underlying parameter estimates. All analyses were performed by using DATA 3.5 (TreeAge Software, Inc, Williamstown, MA).

### Clinical Data

Table 1 summarizes the parameter estimates and their plausible ranges. We assumed that antibiotics used to prevent surgical site infection were partially protective only against infections caused by susceptible bacteria. Vancomycin-susceptible bacteria included all gram-positive organisms except vancomycin-resistant enterococci. Cefazolin-susceptible bacteria included aerobic gram-positive organisms (except enterococci, MRSA, and methicillin-resistant coagulase-negative staphylococci) as well as some aerobic gram-negative bacteria. We based the proportion of surgical site infections attributed to specific causative organisms on microbiologic data from two published studies of patients undergoing CABG surgery, most of whom received a first-generation cephalosporin for prophylaxis (12,13).

The efficacy of antibiotic prophylaxis in patients undergoing CABG surgery is difficult to quantify directly since the only available placebo-controlled studies were terminated early because significantly better outcomes occurred in the patients assigned to prophylaxis compared with controls (34,35). Therefore, we assumed a relative risk of 0.4 for a surgical site infection in patients who received antibiotic prophylaxis compared with those who did not, which corresponds to the highest effectiveness of antibiotic prophylaxis within the range of results from completed placebo-controlled studies in clean surgical procedures other than CABG surgery (18-23). We used data on the incidence of surgical site infections among patients receiving cefazolin from five surveillance programs in university-affiliated hospitals in Boston (17). By assuming that patients who received either cefazolin or vancomycin shared the same relative risk of 0.4 of developing a surgical site infection due to a susceptible organism, compared with patients who did not receive prophylaxis, we were able to estimate the incidence of surgical site infections for each strategy. We used data from the National Nosocomial Infection Surveillance System (26) to estimate the proportion of surgical site infections caused by antibiotic-resistant organisms. We recognize that the incidence of surgical site infections caused by antibiotic-resistant organisms varies from one institution to another, and therefore varied the resistance pattern over a wide range in sensitivity analysis.

We assumed that 10% of patients had a history of allergy to beta-lactam antibiotics (27). The incidence of adverse events secondary to vancomycin was based on data from a prospective study in which vancomycin was used for 10 days (24). We adjusted these estimates to reflect the probability of toxicity with a 2-day prophylactic regimen by assuming a linear relationship between the incidence of adverse events and the duration of therapy. We assumed that the incidence of adverse events was the same for cefazolin and vancomycin, since several comparative prophylactic studies have reported the toxicity profiles to be similar (25,36,37). Death rates secondary to deep surgical site infection (12), and anaphylactic reaction to cefazolin (28) were obtained from published studies. We then derived the deaths associated with the surgical procedure and its noninfectious complications by using data on all-cause hospital deaths following CABG surgery reported for the state of Massachusetts (29).

We estimated quality-adjusted life expectancy by applying quality weights to the health states representing death, myocardial infarction, angina, or asymptomatic coronary artery disease. These quality weights were obtained from the Beaver Dam Health Outcomes Study, in which time trade-off techniques were used to elicit utilities (38). We explored a wide range of quality-weights for the temporary health states reflecting a superficial or deep surgical site infection.

### Costs

To estimate the costs associated with surgical site infections and hospital deaths, we relied on published estimates (30-33) and adjusted these to 1998 U.S. dollars by using the medical care component of the consumer price indexes published by the Bureau of Labor Statistics (39). These studies used costs from a cost accounting system (30,32) or charges

Table 1. Model variables[a]

| Variables | Base case | Plausible range | References |
|---|---|---|---|
| **Incidence of SSI** | | | |
| Superficial | 0.08 | 0.02 – 0.12 | (12,17) |
| Deep | 0.04 | 0.01 – 0.06 | (12,17) |
| **Causative organisms** | | | (12,13) |
| *Staphylococcus aureus* | 0.25 | 0.20 – 0.35 | |
| Coagulase-negative *Staphylococci* | 0.25 | 0.20 – 0.35 | |
| Enterococci | 0.05 | 0.02 – 0.15 | |
| Gram-negative bacteria | 0.30 | 0.15 – 0.50 | |
| **Relative risk of SSI caused by susceptible organisms** | | | |
| Vancomycin vs. no prophylaxis | 0.4 | 0.20 – 0.80 | (18-23) |
| Cefazolin vs. no prophylaxis | 0.4 | 0.20 – 0.80 | (18-23) |
| **Incidence of antibiotic-related adverse events** | | | |
| Vancomycin | 0.08 | 0.01 – 0.20 | (24) |
| Cefazolin | 0.08 | 0.01 – 0.20 | (24,25) |
| **Incidence of SSI due to resistant organisms** | | | (26) |
| MRSA (% of all SSI due to *S. aureus)* | 0.012 (0.40) | 0 – 0.03 | |
| Methicillin-resistant CNS (% of all SSI due to CNS) | 0.024 (0.80) | 0 – 0.03 | |
| VRE (% of all SSI due to enterococci) | 0.003 (0.15) | 0 – 0.006 | |
| Incidence of SSI caused by cefazolin-susceptible gram-negative bacteria (% of all SSI due to gram-negative bacteria) | 0.01 (0.28) | 0 – 0.036 | |
| History of allergy to beta-lactams | 0.1 | 0.05 – 0.15 | (27) |
| **Probability of hospital death** | | | |
| Deep surgical site infection | 0.082 | 0.01 – 0.10 | (12) |
| Antibiotic allergic reaction | 0.00002 | | (28) |
| Coronary artery bypass graft surgery-related events | 0.036 | 0.01 – 0.1 | (29) |
| **Costs per case (US$)** | | | |
| Vancomycin | 80 | 60 – 250 | (24) |
| Cefazolin | 24 | 10 – 50 | (24) |
| Superficial SSI | 8,000 | 3000 – 15,000 | (30,31) |
| Deep SSI | 36,800 | 10,000 – 50,000 | (30,31) |
| Vancomycin-related adverse event | 107 | 20 – 1,000 | (24) |
| Cefazolin-related adverse event | 107 | 20 – 1,000 | (24) |
| Death | 5,900 | 0 – 10,000 | (32) |
| Multiplication factor for infections due to methicillin-resistant organisms | 1.13 | 0.9 – 2 | (33) |
| Multiplication factor for infections due to VRE | 1.5 | 0.9 – 3 | [b] |

[a]SSI = surgical site infection; MRSA = methicillin-resistant *Staphylococcus aureus*; CNS = coagulase-negative staphylococci; VRE: vancomycin-resistant enterococci.
[b]We assumed that the cost of an infection caused by a vancomycin-resistant enterococcus was 50% greater than the cost of a comparable infection caused by a susceptible strain.

converted to costs (31,33) as a proxy for direct medical costs. We based the costs of prophylaxis-related adverse events on a study of vancomycin (24) and assumed identical costs for cefazolin-related adverse events. We assumed that both vancomycin and cefazolin would be used for 48 hours, which implies a total of 5 doses of 1 g of vancomycin or 6 doses of 1 g of cefazolin. Antibiotic costs were based on hospital pharmacy acquisition costs, although we added the cost associated with perfusion for both antibiotics (24). A preparation cost was added for vancomycin only, since cefazolin is available in bags ready for infusion.

We estimated the costs of follow-up care by extrapolating the 5-year follow-up medical care cost after CABG surgery among patients included in the Bypass Angioplasty Revascularization Investigation (40).

### Results

### Base Case

Table 2 shows the intermediate health outcomes and costs associated with the three prophylactic strategies for a hypothetical cohort of 10,000 patients undergoing CABG surgery. With no prophylaxis, the model predicted 570 deep surgical site infections, 1,141 superficial surgical site infections, and 405 all-cause hospital deaths. Prophylaxis using routine cefazolin, reserving vancomycin for patients with a history of beta-lactam allergy, resulted in 173 fewer deep surgical site infections, 347 fewer superficial surgical site infections, and 14 fewer deaths compared with no prophylaxis. Prophylaxis using routine vancomycin resulted in 29 fewer deep surgical site infections, 58 fewer superficial surgical site infections, and 3 fewer deaths, compared with routine cefazolin. Routine vancomycin use was also associated with the lowest direct medical costs for a 3-month period and cost $1,170,000 less than routine cefazolin strategy per 10,000 patients. Since the routine vancomycin strategy was more effective and less costly, the strategy of routine cefazolin was eliminated by strong dominance.

### Sensitivity Analysis

A strategy of no prophylaxis was always less effective and more costly than using prophylaxis. Ranking of the routine vancomycin and cefazolin strategies, both in terms of costs and clinical outcomes, was not affected when the following parameters were changed over the plausible range described in Table 1: deaths from all causes and surgical site infection-related deaths; incidence of deep or superficial surgical site infection; distribution of causative organisms; incidence of prophylaxis-related adverse events; proportion of patients with allergy to beta-lactam antibiotics; costs of cefazolin, deep or superficial surgical site infections, death, or prophylaxis-related adverse events.

Results were most sensitive to changes in the cost of vancomycin, efficacy of cefazolin and vancomycin in preventing surgical site infections, and prevalence of bacterial resistance to antibiotics. If the acquisition and administration cost of 5 doses of vancomycin exceeded a threshold of $215, cefazolin was no longer dominated by vancomycin since routine vancomycin became more costly. Similarly, routine vancomycin became more costly than routine cefazolin if vancomycin prevented 18% fewer infections caused by susceptible organisms compared with cefazolin; if this difference exceeded 25%, the routine vancomycin strategy was less effective and more costly and was thus dominated by the cefazolin strategy.

We explored the impact of different antibiotic susceptibility profiles, as might be observed in different hospitals, on these results. Routine vancomycin remained the most effective and the least costly strategy independent of the prevalence of vancomycin-resistance in enterococci. Figure 2 shows a three-way sensitivity analysis of the incidence of surgical site infection caused by each of the following pathogens: an MRSA; a methicillin-resistant coagulase-negative staphylococcus; and a cefazolin-susceptible gram-negative bacteria. For a given incidence of surgical site infection caused by MRSA, each line represents the threshold combinations of methicillin resistance in coagulase-negative staphylococci and cefazolin susceptibility in gram-negative bacteria for routine vancomycin to be more cost-effective than routine cefazolin. For example, in the base case, we assumed a 2.4 per 100 risk for infection caused by methicillin-resistant coagulase-negative staphylococci and a 1.0 per 100 risk for surgical site infection caused by cefazolin-susceptible gram-negative bacteria. Routine cefazolin was more cost-effective than vanco-

Table 2. Base-case analysis: clinical outcomes and costs for a hypothetical cohort of 10,000 patients undergoing coronary artery bypass graft surgery

| Strategy | Deep SSI | Increm. deep SSI[a] | Superficial SSI | Increm. superficial SSI[a] | Hospital deaths | Increm. hospital deaths[a] | Deaths, SSI, or both | Increm. deaths, SSI, or both | Costs (x $1,000) | Increm. costs (x $1,000)[a] |
|---|---|---|---|---|---|---|---|---|---|---|
| No prophylaxis | 570 | - | 1141 | - | 405 | - | 2,008 | - | 33,410 | - |
| Routine cefazolin | 397 | - 173[b] | 794 | - 347[b] | 391 | - 14[b] | 1,506 | - 502[b] | 24,530 | - 8,880[b] |
| Routine vancomycin | 368 | - 29[b] | 736 | - 58[b] | 388 | - 3[b] | 1,423 | - 83[b] | 23,360 | - 1,170[b] |

SSI = surgical site infection; increm = incremental.

[a]Routine cefazolin compared with no prophylaxis; routine vancomycin compared with routine cefazolin.

[b]Negative incremental numbers of infections or deaths represent the numbers of infections or deaths averted; negative incremental costs represent costs saving.

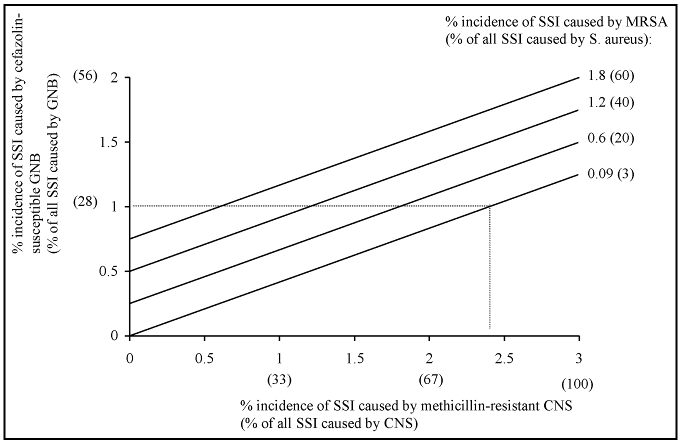

Figure 2. Three-way sensitivity analysis of the incidence of surgical site infection (SSI) caused by methicillin-resistant *Staphylococcus aureus* (MRSA); methicillin-resistant coagulase-negative staphylococci; or cefazolin-susceptible gram-negative bacteria. The lines show the incidence of infection caused by methicillin-resistant *Staphylococcus aureus* necessary for routine cefazolin prophylaxis to be more cost-effective than routine vancomycin (0.09%, 0.6%, 1.2%, and 1.8%). For a particular line, points to the lower right indicate that routine vancomycin is more cost-effective; points to the upper left indicate that routine cefazolin is more cost-effective. The dotted line represents the example cited in text. SSI = surgical site infection; MRSA = methicillin-resistant *Staphylococcus aureus;* CNS = coagulase-negative staphylococci; GNB = gram-negative bacteria.

mycin only when the incidence of surgical site infections caused by MRSA was lower than 0.09 per 100, which represents 3% of the infections caused by *S. aureus*.

The impact of different patterns of antimicrobial resistance on the incremental cost-effectiveness ratio associated with routine vancomycin compared with routine cefazolin is shown in Figure 3. This ratio represents the added cost of using vancomycin divided by the additional clinical benefits provided by vancomycin (i.e., additional death or surgical site infection averted), compared with the next least expensive strategy. For example, in a hypothetical hospital where MRSA caused surgical site infections in 1% of the patients undergoing CABG surgery, methicillin-resistant coagulase-negative staphylococci in 2.5%, and cefazolin-susceptible bacteria in 1.5%, the incremental cost-effectiveness ratio for routine vancomycin was $10,237 per additional infections or death averted, compared with routine cefazolin.

### Reference Case

In a hypothetical cohort of 65-year-old men undergoing CABG surgery, quality-adjusted life expectancy was 8.312 quality-adjusted life-years, and per person lifetime costs were $62,892 in the absence of prophylaxis (Table 3). A strategy of prophylaxis with routine cefazolin was $876 less costly and provided an additional 0.023 quality-adjusted life years compared with no prophylaxis. The most effective strategy, prophylaxis with routine vancomycin, saved an additional $103 compared with cefazolin and therefore dominated a strategy of routine cefazolin.

Similar to the results for the base case, our reference case results were most sensitive to estimates for the acquisition and administration cost of vancomycin; the efficacy of vancomycin and cefazolin in preventing surgical site infections; and the prevalence of bacterial resistance to antibiotics.

### Discussion

In the base-case analysis, a strategy of routine vancomycin prophylaxis in the overall population of CABG patients was more effective than a strategy of routine cefazolin, since it prevented more surgical site infections or deaths caused by methicillin-resistant staphylococci or enterococci. Routine vancomycin was also less costly than cefazolin, an advantage that was offset neither by higher acquisition and administration costs nor by the absence of protection against gram-negative bacteria. Although these results were dependent on the prevalence of antibiotic resistance in the causative organisms, the range of circumstances in which a strategy of routine cefazolin was either more effective or less costly than vancomycin was narrow, restricted to situations in which MRSA represented no more than 3% of all *S. aureus*.

In the reference case analysis, a strategy of routine vancomycin was also more effective and less costly than a strategy of routine cefazolin. Because of a lack of available data, we were not able to quantify the precise contribution of routine vancomycin use to the development of vancomycin-resistance in gram-positive organisms. However, we did conduct sensitivity analyses to explore the impact of a decrease in efficacy of vancomycin on our results. Estimating the future economic consequences that might be associated with the development of vancomycin resistance is more difficult. Antibiotic resistance, described in economic terminology as an externality associated with the use of antimicrobials (41), is an effect of antimicrobial use that is unlikely to be felt by either the patient or the provider. We know from experience with vancomycin-resistant enterococci that surveillance programs, isolation of colonized patients, and treatment of infections that resist most existing antibiotics increase costs.

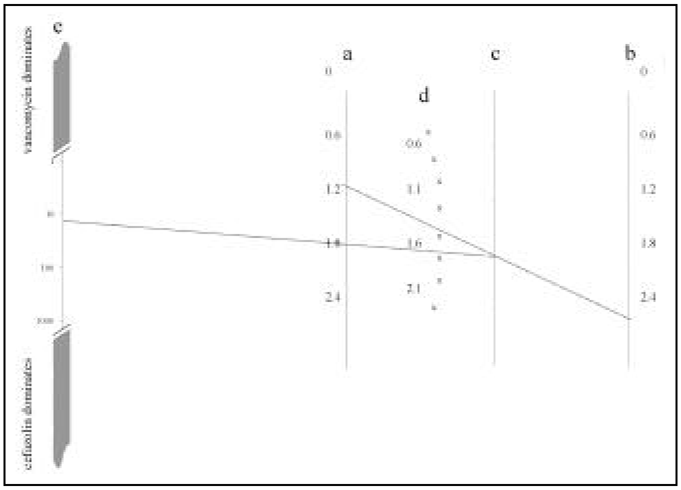

Figure 3. Determination of the incremental cost-effectiveness ratio of the routine vancomycin strategy relative to the routine cefazolin strategy, according to bacterial resistance pattern. To determine the incremental cost-effectiveness ratio: 1) Place percent incidence of surgical site infection caused by methicillin-resistant *Staphylococcus aureus* on the a axis; 2) Place percent incidence of surgical site infection caused by methicillin-resistant coagulase-negative staphylococci on the b axis; 3) Draw a line between these 2 points. This line crosses the c axis at a point x; 4) Place percent incidence of surgical site infection caused by cefazolin-susceptible gram-negative bacteria on the scale d; 5) Draw a line passing through this point and the point x on the c axis. If this line crosses the e axis between the two zones of dominance, the incremental cost-effectiveness ratio can be red (in thousands of dollars per additional death or surgical site infection averted). The dotted lines represent the example cited in text.

Table 3. Reference case analysis: quality-adjusted life expectancy and lifetime costs for a 65-year-old man undergoing coronary artery bypass graft surgery

| Strategy | Total costs ($) | Incremental costs[a] ($) | QALYs | Incremental QALYs[a] | Incremental cost-effectiveness ratio[a] |
|---|---|---|---|---|---|
| No prophylaxis | 62,892 | - | 8.312 | - | - |
| Routine cefazolin | 62,016 | - 876[b] | 8.335 | 0.023 | Dominated[c] |
| Routine vancomycin | 61,913 | - 103[b] | 8.339 | 0.004 | Cost saving |

QALYs = quality-adjusted life years.
[a]Routine cefazolin compared with no prophylaxis; routine vancomycin compared with routine cefazolin.
[b]Negative incremental costs represent cost savings.
[c]A dominated strategy is one that costs more and is less effective than an alternative strategy.

Similarly, the spread of vancomycin resistance in highly pathogenic species such as *S. aureus* could have substantial clinical and economic consequences. When data become available to document and quantify the relationship between the routine use of vancomycin and vancomycin resistance, we will be able to better describe the trade-off between the short-term benefits to individual patients and the long-term consequences to society at large. However, there are similar uncertainties with respect to the consequences of routine cefazolin, which selects for MRSA and cefazolin-resistant gram-negative organisms and is a recognized risk factor for the acquisition of infection caused by vancomycin-resistant enterococci (4).

We believe our decision not to model the relationship between antibiotic prophylaxis and resistance had a limited impact on our results. For instance, routine vancomycin would still be more effective and less costly than routine cefazolin if all enterococci were resistant to vancomycin because of the small proportion of surgical site infections caused by enterococci after CABG surgery. The spread of vancomycin resistance in staphylococci, however, would impact the model more substantially. We therefore simulated a hypothetical scenario in which prevalence of vancomycin resistance in enterococci would continue to increase by about 2% per year, as reported in U.S. hospitals from 1989 through 1997 (4), and in which the same trend would be observed in staphylococci (data not shown). We arbitrarily assumed that vancomycin prophylaxis, but not cefazolin prophylaxis, would accelerate this trend by 50%. Under these circumstances, routine vancomycin would no longer be less costly than routine cefazolin after 6 years and would also become less effective after 13 years. Although such a simulation addresses the issue of future resistance crudely and incompletely, it illustrates how speculative it would be to model this evolution, given the current lack of knowledge about glycopeptide resistance in staphylococci.

The conclusions of this analysis were not meaningfully influenced by uncertainty around the model parameters, according to sensitivity analyses conducted over a wide range of plausible values. Other than the influence of susceptibility patterns discussed above, the results were sensitive only to large variations of the price of vancomycin and to the relative effectiveness of the two prophylactic drugs. This last aspect is a limitation of the study due to insufficient data. The effectiveness of cefazolin may have been underestimated if cefazolin has some effect against methicillin-resistant staphylococci when the inoculum is small, as may be the case during surgery. We can also speculate that a large proportion of methicillin-resistant organisms are acquired after surgery; perioperative antibiotic prophylaxis may not prevent infections caused by these organisms. We are not aware of data supporting this hypothesis, however. We assumed that both vancomycin and cefazolin had the same preventive efficacy against susceptible organisms. However, vancomycin would no longer be the best option if it were 18% less effective than cefazolin in preventing infections caused by susceptible organisms. Beside uncertainty regarding the relative intrinsic effectiveness of the two drugs, this effectiveness may be differently affected by suboptimal use such as wrong timing or wrong dose, because of distinct pharmacokinetic characteristics. In general, vancomycin's longer half-life in serum would be expected to make it more tolerant than cefazolin if delays occur between administration of prophylaxis and initiation of surgery. Finally, we did not include patient time costs in the reference case analysis. However, their inclusion would only have increased the estimated benefit associated with preventing infections.

These results underscore the importance of using a perioperative prophylaxis regimen with reasonable activity against gram-positive organisms. Failure to provide patients undergoing CABG with an acceptable perioperative prophylaxis regimen is an example of a medical error; in the aggregate, these errors have been recognized to negatively affect clinical outcomes and to impose a large burden on health resources (42). Because the circumstances in which perioperative prophylaxis must be administered are highly structured, development of systems to achieve near-perfect compliance could be feasible for the health- care delivery system. The motivation for making this a priority is particularly strong because prophylaxis was estimated to save at least $900 per person. The strategy of no prophylaxis was always associated with both higher costs and a greater number of deaths and surgical site infections than either of the two alternative prophylactic strategies. In fact, the cost attributed to failure to provide prophylaxis may have been underestimated, since we assumed that the risk reduction was similar to that observed in other clean surgical procedures (18-23).

Approximately 366,000 CABG operations are performed yearly in the United States (43). Using the best data currently available and considering clinical outcomes to individual patients, our model predicts that routine vancomycin would prevent 110 deaths and 3,184 surgical site infections compared with routine cefazolin. Under conditions similar to those in our base case, the routine vancomycin strategy would also save $43 million nationwide. Similar conclusions are to be expected from the analysis of clean surgical procedures other than CABG, since most surgical site infections are caused by staphylococci in these settings. Because data are insufficient to provide information about the potential downstream societal consequences of routine vancomycin use on the development of vancomycin resistance, we are reluctant to recommend the universal routine use of vancomycin. However, the incremental clinical benefits and cost savings associated with routine vancomycin compared with routine cefazolin for perioperative prophylaxis in CABG surgery provide an estimate of the magnitude of benefits that would need to result, at least for society at large, from slowing the emergence of vancomycin resistance by restricting its use. We recommend that immediate priority be given to studies that will inform the impact of vancomycin use on the development of resistance in gram-positive organisms.

### Acknowledgments

We thank Fred C. Tenover, Mervin Shapiro, and Stephan Harbarth for their thoughtful comments and advice about this manuscript.

This research was supported in part by cooperative agreement UR8/CCU115079 from the Centers for Disease Control and Prevention, Atlanta, GA.

Dr. Zanetti is supported by grants from the University Hospital of Lausanne and from the Leenaards Foundation, Lausanne, Switzerland.

Dr. Zanetti is an associate hospital epidemiologist and attending physician in infectious diseases at the Lausanne University Hospital, Lausanne, Switzerland. He is also a visiting scientist at the Channing Laboratory, Brigham and Women's Hospital, Boston, MA. His main fields of research are optimization of antibiotics use, surgical site infection, and infection in cancer and intensive-care unit patients.

### References

1. Waldvogel FA. New resistance in *Staphylococcus aureus*. N Engl J Med 1999;340:556-7.
2. Smith TA, Pearson ML, Wilcox KR, Cruz C, Lancaster MV, Robinson-Dunn B, et al. Emergence of vancomycin resistance in *Staphylococcus aureus*. N Engl J Med 1999;340:493-501.
3. Jarvis WR. Epidemiology, appropriateness, and cost of vancomycin use. Clin Infect Dis 1998;26:1200-3.
4. Martone WJ. Spread of vancomycin-resistant enterococci: why did it happen in the United States? Infect Control Hosp Epidemiol 1998;19:539-45.
5. Centers for Diseases Control and Prevention. Recommendations for preventing the spread of vancomycin resistance. Recommendations of the Hospital Infection Control Practices Advisory Committee. MMWR Morb Mortal Wkly Rep 1995;44:1-13.
6. Woods RK, Dellinger EP. Current guidelines for antibiotic prophylaxis of surgical wounds. Am Fam Physician 1998;57:2731-40.
7. Dellinger EP, Gross PA, Barrett TL, Krause PJ, Martone WJ, McGowan JE Jr, et al. Quality standard for antimicrobial prophylaxis in surgical procedures. Clin Infect Dis 1994;18:422-7.
8. Page CP, Bohnen JM, Fletcher JR, McManus AT, Solomkin JS, Wittmann DH. Antimicrobial prophylaxis for surgical wounds. Guidelines for clinical care. Arch Surg 1993;128:79-88.
9. Kaiser AB. Antimicrobial prophylaxis in surgery. N Engl J Med 1986;315:1129-38.
10. ASHP Commission on Therapeutics. ASHP therapeutic guidelines on antimicrobial prophylaxis in surgery. Clin Pharm 1992;11:483-513.
11. Martin C, the French Study Group on Antimicrobial Prophylaxis in Surgery, The French Society of Anesthesia and Intensive Care. Antimicrobial prophylaxis in surgery: general concepts and clinical guidelines. Infect Control Hosp Epidemiol 1994;15:463-71.
12. L'Ecuyer PB, Murphy D, Little JR, Fraser VJ. The epidemiology of chest and leg wound infections following cardiothoracic surgery. Clin Infect Dis 1996;22:424-9.
13. Mossad SB, Serkey JM, Longworth DL, Cosgrove DM, Gordon SM. Coagulase-negative staphylococcal sternal wound infections after open heart operations. Ann Thorac Surg 1997;63:395-401.
14. Gold MR, Siegel JE, Russell LB, Weinstein MC, editors. Cost-effectiveness in health and medicine. New York: Oxford; 1996.
15. Kim C, Kwok YS, Saha S, Redberg RF. Diagnosis of suspected coronary artery disease in women: a cost-effectiveness analysis. Am Heart J 1999;137:1019-27.
16. Peduzzi P, Kamina A, Detre K. Twenty-two year follow-up in the VA cooperative study of coronary artery bypass surgery for stable angina. Am J Cardiol 1998;81:1393-9.
17. Sands K, Yokoe D, Hooper D, Tully J, Platt R. A multi-institutional comparison of surgical site infection surveillance by screening of administrative and pharmacy data. Proceedings of the 8th Annual Meeting of the Society for Healthcare Epidemiology of America, San Francisco, CA, 1999. Thorofare (NJ):Slack Inc; 2000. Abstract M35.
18. Platt R, Zaleznik DF, Hopkins CC, Dellinger EP, Karchmer AW, Bryan CS, et al. Perioperative antibiotic prophylaxis for herniorrhaphy and breast surgery. N Engl J Med 1990;322:153-60.
19. Kaiser AB, Clayson KR, Mulherin JL, Roach AC, Allen TR, Edwards WH, et al. Antibiotic prophylaxis in vascular surgery. Ann Surg 1978;188:283-189.
20. Platt R, Zucker JR, Zaleznick DF, Hopkins CC, Dellinger EP, Karchmer AW, et al. Prophylaxis against wound infection following herniorrhaphy or breast surgery. J Infect Dis 1992;166:556-60.
21. Bold RJ, Mansfield PF, Berger DH, Pollock RE, Singletary SE, Ames FC, et al. Prospective, randomized, double-blind study of prophylactic antibiotics in axillary lymph node dissection. Am J Surg 1998;176:239-43.
22. Platt R, Zucker JR, Zaleznick DF, Hopkins CC, Dellinger EP, Karchmer AW, et al. Perioperative antibiotic prophylaxis and wound infection following breast surgery. J Antimicrob Chemother 1993;31 Suppl B:43-8.
23. Bencini PL, Galimberti M, Signorini M, Crosti C. Antibiotic prophylaxis of wound infections in skin surgery. Arch Dermatol 1991;127:1357-60.
24. Garrelts JC, Horst WD, Silkey B, Gagnon S. A pharmacoeconomic model to evaluate antibiotic costs. Pharmacotherapy 1994;14:438-45.
25. Maki DG, Bohn MJ, Stolz SM, Kroncke GM, Acher CW, Myerowitz PD. Comparative study of cefazolin, cefamandole, and vancomycin for surgical prophylaxis in cardiac and vascular operations. J Thorac Cardiovasc Surg 1992;104:1423-34.
26. Centers for Disease Control and Prevention. National Nosocomial Infection Surveillance System. NNIS antimicrobial resistance surveillance report. 1999. Available from: URL: http://www.cdc.gov/ncidod/hip/NNIS/AR_Surv1198.htm
27. Condemi JJ, Sheehan MG. Allergy to penicillin and other antibiotics. In: Reese RE, Betts RF, editors. A practical approach to infectious diseases. 4th ed. Boston: Little, Brown and Co.; 1996. p. 1037-58.
28. Idsol O. Nature and extent of penicillin side reactions with particular reference to fatalities from anaphylactic shock. Bull World Health Organ 1968;38:159.
29. Ghali WA, Ash AS, Hall RE, Moskowitz MA. Statewide quality improvement initiatives and mortality after cardiac surgery. JAMA 1997;277:379-82.
30. VandenBergh MFQ, Kluytmans JAJW, van Hout BA, Maat APWN, Seerden RJ, McDonnel J, et al. Cost-effectiveness of perioperative mupirocin nasal ointment in cardiothoracic surgery. Infect Control Hosp Epidemiol 1996;17:786-92.

31. Zoutman D, McDonald S, Vethanayagan D. Total and attributable costs of surgical-wound infections at a canadian tertiary-care center. Infect Control Hosp Epidemiol 1998;19:254-9.
32. Hall RE, Ash AS, Ghali WA, Moskowitz MA. Hospital cost of complications associated with coronary artery bypass graft surgery. Am J Cardiol 1997;79:1680-2.
33. Rubin RJ, Harrington CA, Poon A, Dietrich K, Greene JA, Moiduddin A. The economic impact of *Staphylococcus aureus* infection in New York City hospitals. Emerg Infect Dis 1999;5:1-12.
34. Penketh AR, Wansbrough-Jones MH, Wright E, Pepper JR, Parker DJ. Antibiotic prophylaxis for coronary artery bypass graft surgery. Lancet 1985;1:1500.
35. Fong IW, Baker CB, McKee DC. The value of prophylactic antibiotics in aorto-coronary bypass operations: a double-blind randomized trial. J Thorac Cardiovasc Surg 1979;78:908-13.
36. Suter F, Avai A, Gerundini M, Caprioli S, Maggiolo F. Teicoplanin versus cefamandole in the prevention of infection in total hip replacement. Eur J Clin Microbiol Infect Dis 1994;13:793-6.
37. Vuorisalo S, Pokela R, Syrjälä H. Comparison of vancomycin and cefuroxime for infection prophylaxis in coronary artery bypass surgery. Infect Control Hosp Epidemiol 1998;19:234-9.
38. Fryback DG, Dasbach EJ, Klein R, Klein BEK, Dorn N, Peterson K, et al. The Beaver Dam health outcomes study: initial catalog of health-state quality factors. Med Decis Making 1993;13:89-102.
39. Bureau of Labor Statistics. Consumer price indexes. 1999. Available from: URL: http://stats.bls.gov/cpihome.htm
40. Hlatky MA, Rogers WJ, Johnstone I, Boothroyd D, Brooks MM, Pitt B, et al. Medical care costs and quality of life after randomization to coronary angioplasty or coronary bypass surgery. N Engl J Med 1997;336:92-9.
41. Coast J, Smith RD, Millar MR. An economic perspective on policy to reduce antimicrobial resistance. Soc Sci Med 1998;46:29-38.
42. The National Academies, Institute of Medicine. To err is human: building a safer health system. 1999. Available from: URL: http://www4.nationalacademies.org/news.nsf
43. Lawrence L, Hall MJ. National Center for Health Statistics. 1977 summary: national hospital survey. Advance Data 1999;308:1-16.
